# Kukoamine A attenuates lipopolysaccharide-induced apoptosis, extracellular matrix degradation, and inflammation in nucleus pulposus cells by activating the P13K/Akt pathway

**DOI:** 10.1080/21655979.2022.2051855

**Published:** 2022-03-25

**Authors:** Dan Wang, Hao Qu, Hui Kang, Feng Xu, Wei Huang, Xianhua Cai

**Affiliations:** a College of Acupuncture and Orthopedics, Hubei University of Chinese Medicine, Wuhan, China; b Department of Spine Surgery, Jinmen NO. 2 People’s Hospital, Jingmen, China; c Department of Orthopedics Surgery, PLA Middle Military Command General Hospital, Wuhan, China; d Department of Orthopaedics, Hubei Provincial Hospital of Integrated Chinese & Western Medicine, Wuhan, China

**Keywords:** Kukoamine A, NPCs, P13K/Akt pathway, intervertebral disc degeneration

## Abstract

Intervertebral disc degeneration (IDD) is the leading cause of back, neck, and radicular pain. This study aims to look at the roles of Kukoamine A (KuA) in nucleus pulposus cells (NPCs) of IDD and its related potential mechanisms. Cell viability of NPCs in the control, lipopolysaccharide (LPS) and LPS+KuA groups was firstly detected by cell counting kit (CCK)-8. Meanwhile, the protein expression of collagen II in LPS-induced NPCs was measured by western blot. Then, the experiments following the treatment of KuA in LPS-induced NPCs included cell proliferation assessment by 5-ethynyl-2’-deoxyuridine (EdU) kit, cell apoptosis and extracellular matrix degradation (ECM) analysis by Terminal dUTP nick-end labeling (TUNEL) and western blot, the detection of inflammatory cytokines by western blot and enzyme-linked immunosorbent assay (ELISA), P13K/Akt pathway-related protein levels analysis by western blot. Finally, after the addition of P13K/Akt pathway inhibitor LY294002, cell apoptosis, ECM and inflammation in KuA-treated NPCs induced by LPS were again examined by the same methods. Results indicated that KuA prevented loss of cell viability and attenuated the apoptosis, ECM, and inflammation in LPS-induced NPCs. Furthermore, western blot experiment verified the activation of KuA on P13K/Akt pathway in LPS-induced NPCs. However, inhibition of P13K/Akt pathway reversed the roles of KuA in LPS-induced NPCs. Thus, KuA attenuates LPS-induced apoptosis, ECM and inflammation in LPS-induced NPCs by activating the P13K/Akt pathway.

## graphical abstract

**Figure:**
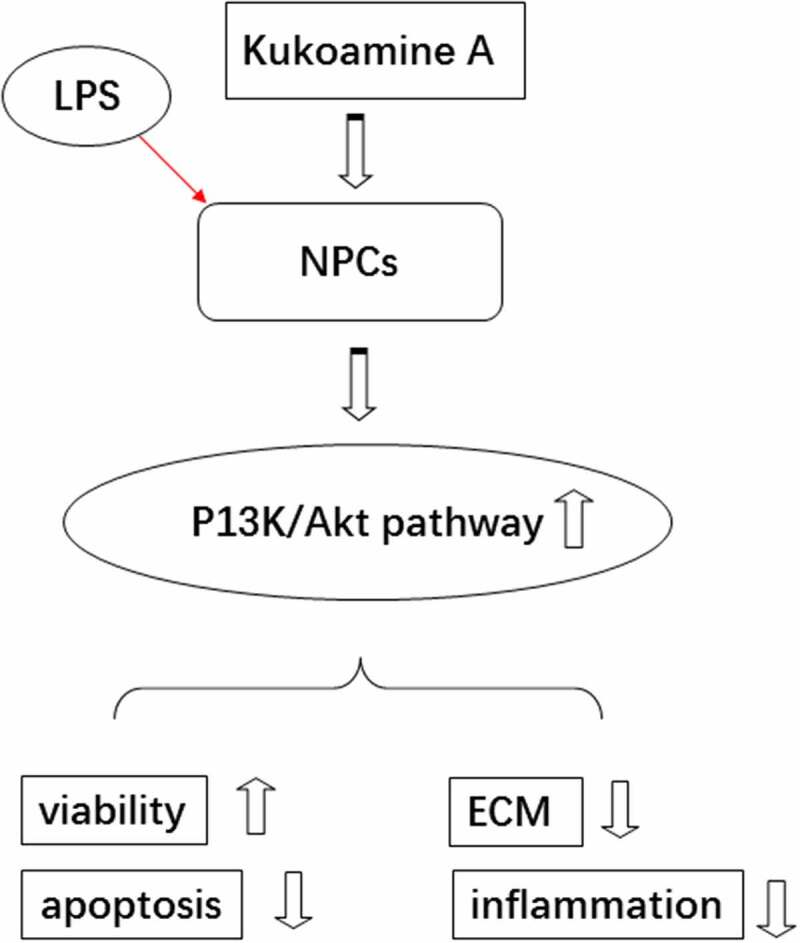

## Introduction

Intervertebral disc degeneration (IDD) is a progressive functional and structural damage resulting from cellular and biochemical changes in the intervertebral disc microenvironment [1,2]. It is also the leading cause of back, neck, and radicular pain [3]. It has been well documented that 40% of cases of low back pain and 90% of cases of sciatica are associated with IDD [4,5]. A large body of literature suggests that genetic predisposition, age, lifestyle (obesity, smoking, and depressive symptoms) and non-physiological mechanical loading are the main causes of IDD [^6–8^]. Numerous studies have analyzed IDD from the perspectives of degeneration and regeneration, mechanics and biology [9,10]. But, a systematic understanding of intrinsic cellular changes is still lacking. Some studies have shown that IDD is closely associated with an inflammatory response [9]. During degeneration, a number of cytokines can promote matrix degradation, chemokine production, and lead to changes in cellular phenotype [3]. In addition, apoptosis of nucleus pulposus cells (NPCs) also plays a key role in intervertebral disc degeneration [11]. Up to the present time, although treatments for IDD, such as gene therapy, growth factor injections, tissue engineering, and cell-based therapies provide improvement in its degeneration, it is still more challenging to restore IDD to a healthy state [12]. Thus, there is an extremely urgent need to find new therapies and drugs for IDD.

Kukoamine A (KuA) is an arginine alkaloid with bioactive components extracted from the root bark of Lycium Chinense [13]. Previous studies have confirmed that KuA is capable of a variety of pharmacological effects, such as anti-inflammatory, antioxidant, and anti-apoptotic effects. For example, KuA was reported to markedly attenuate H_2_O_2_-induced apoptosis in SHSY5Y cells by inhibiting oxidative stress and inactivating apoptotic pathways [14]. KuA exerts a protective effect against radiation brain injury via inhibiting oxidative stress and neuronal apoptosis [15]. However, the role and the potential mechanism of KuA in IDD are still unclear.

P13K/Akt is a cellular signaling pathway that regulates growth [16]. Akt can help in cell cycle control and initiation of apoptotic process by interacting with P13K signaling pathway [17]. P13K/Akt also acts as a key regulator in IDD. It has been suggested that the activated PI3K/Akt pathway may provide protection against IDD through multiple mechanisms [18]. For instance, bone morphogenetic protein 2 (BMP2) can mediate extracellular matrix degradation (ECM) and apoptosis in the NPCs to reduce disc degeneration by upregulating PI3K/Akt pathway phosphorylation levels [19]. Moreover, the Apelin-13/APJ system can activate the PI3K/Akt signaling pathway to delay disc degeneration [20]. Additionally, pretreatment with KuA may promote the PI3K/Akt/GSK-3β signaling pathway [21]. Accordingly, we hypothesized that KuA may play a protective role in IDD through the PI3K/Akt pathway.

In the current study, we aimed to identify the effects of KuA on LPS-induced NPCs and the potential mechanisms. We hypothesized that KuA could alleviate LPS-induced injury in NPCs by activating the P13K/Akt pathway. We first observed the changes in cell viability of NPCs under LPS induction and after treatment with KuA. Subsequently, the apoptosis, outer matrix degradation and inflammation after the treatment of KuA were explored in LPS-induced NPCs, respectively. The data obtained from these experiments provided a new approach and theoretical basis for the treatment of IDD.

## Materials and methods

### Cell culture and treatment

Human nucleus pulposus cells NPCs were provided by Procell Life Science&Technology Co., Ltd. (Wuhan, China) and grown in DMEM with 10% FBS and 1% penicillin-streptomycin. Cells were placed in a suitable incubator with 95% air and 5% CO_2_ at 37°C.

NPCs were divided into the groups of control, LPS, KuA, LPS+KuA. Cells were induced with LPS (0.01, 0.1, 1, 10 µg/ml) in serum-free medium for 24 h. The group without any treatment served as the control. Cells were treated with KuA at the concentration of 10, 20, 40, 80 µM for 4 h. The cells were then harvested for later experiments [22].

### Cell Counting Kit (CCK)-8 assay

NPCs in different groups with or without the treatment of LPS or KuA were kept in 96-well plates at a density of 5 × 10^3^ cells/well for 24 h incubation at 37°C. Then, 10 µl CCK-8 solution (Dojindo, Tokyo, Japan) was added into each well and cultured together with cells for 2 h. Finally, a microplate reader was adopted to measure the OD value at 490 nm [23].

### Western blot assay

Protein extraction from NPCs was carried out with the use of ice-cold RIPA buffer (Beyotime Biotechnology, Shanghai, China). Then, the concentration of the collected protein was assessed by the BCA assay kit (Glpbio, Shanghai, China). Next, proteins were electrophoresed using 12% SDS-PAGE and transferred onto the PVDF membranes carefully. Being blocked with 5% fat-free milk and washed with TBST for 1 h, co-incubation of cells and primary antibody was conducted immediately at 4°C overnight. The primary antibodies used here were against Bcl2, Bax, C-cleaved 3, MMP13, ADAMTS5, Aggrecan, Collagen II, iNOS, COX2, p-P13K, P13K, p-Akt, Akt. Following washing with TBST three times, the membranes were cultured with secondary anti-rabbit antibody in TBST buffer at room temperature for 2 h. An enhanced ECL kit (Thermo Scientific, Waltham, Massachusetts, USA) was applied to visualize the proteins in line with the operating guidelines [24].

### EdU detection

Cell proliferation was determined and quantified by fluorescence microscopy using EdU Staining Proliferation Kit (iFluor 488) (Abcam, Cambridge, MA, USA). Briefly, NPCs were seeded into 96-well plates at a density of 5 × 10^3^ cells/well for 24 h incubation so that they are at 60%–70% confluence. 10 µM EdU solution was prepared to label and culture these cells for 2 h under optimum cell growth conditions. Then, 15 min cell fixation and 20 min permeabilization was performed at room temperature. Subsequently, the reaction mixture of fluorescently labeled EdU was added and incubated for 30 min. EdU positive cells were observed under a fluorescence microscope equipped with an Ex/Em = 491/520 nm filter.

### TUNEL measurement

Cell apoptosis was assessed by the way of Tunel assay [25]. Adherent cells were washed once with PBS. Then, cells were fixed with 4% paraformaldehyde for 30 min. After washing again with PBS, cells were incubated with PBS containing 0.3% Triton X-100 for 2 min at room temperature. One Step Apoptosis Assay Kit (Beyotime Biotechnology, Shanghai, China) was prepared at the appropriate ratio. Subsequently, 50 µl of TUNEL assay solution was added to the samples and incubated for 1 h at 37°C in the dark. After washing 3 times with PBS, the green fluorescing cells were observed by fluorescence microscopy.

### ELISA assay

NPCs were cultured in 24-well plates at a density of 1 × 10^4^/well and cell supernatants were collected. Then, total protein levels contained in each sample were normalized before performing ELISA to measure TNF-α, IL-6 and IL-10. Finally, the levels of TNF-α, IL-6, and IL-10 in the cell supernatant were measured by ELISA assay kit (Shanghai ExCell Biotechnology Co., Ltd.) in keeping with the protocols of the vendor.

### Statistical analysis

All data were recorded and presented by the way of mean ± SD. Data management and analysis were carried out by the use of SPSS version 18.0 (SPSS, Chicago, IL, USA). Significant comparisons were set between two groups using Student’s t-test and multiple groups by one-way ANOVA. A probability of less than 0.05 was indicative of statistically significant data.

## Results

In this study, we studied the effects of KuA and the potential mechanism in LPS-induced NPCs. The results revealed that the KuA rehabilitated the cell viability, suppressed apoptosis, ECM, and inflammatory response in LPS–induced NPCs. In addition, KuA activated the P13K/Akt pathway. Moreover, inhibition of the P13K/Akt pathway reversed the protective effect of KuA on LPS-induced NPCs

### KuA attenuates LPS-induced cell viability damage in NPCs

**Figure 1.**
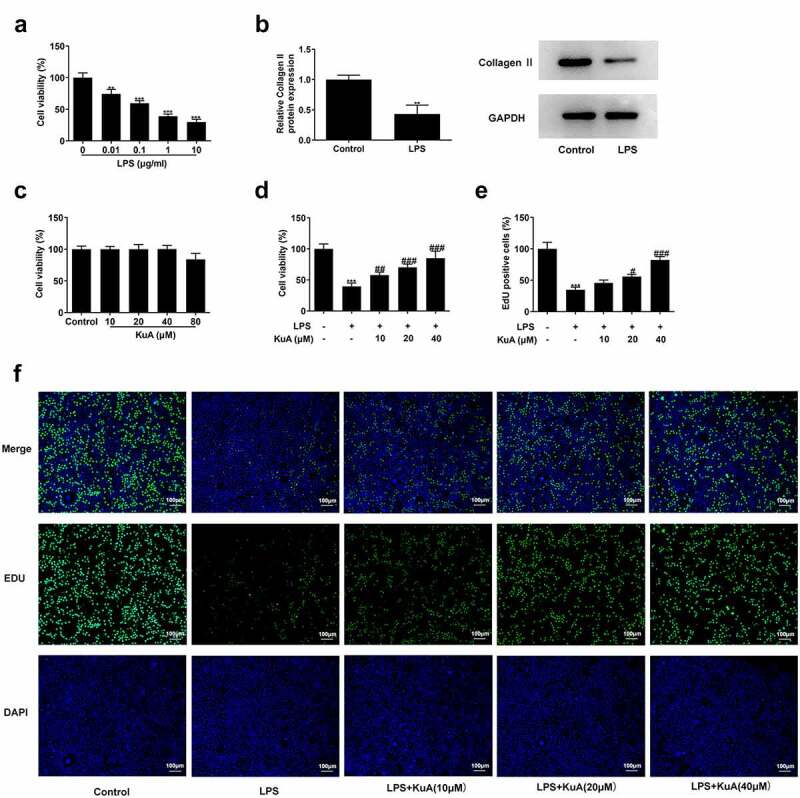
KuA attenuates LPS-induced cell viability damage in NPCs.

To identify the influence of KuA on cell viability in LPS-induced NPCs, the assays of CCK-8, western blot, and EdU were run the cell viability under different conditions. Figure 1a revealed that there has been a marked declined viability in LPS-induced NPCs (vs Control), and cell viability decreased with the increase of LPS concentration. The group with LPS concentration of 1 µg/ml was selected for the follow-up experiment. For further verification of the induction of LPS, the protein concentration of Collagen II was analyzed. Based on the grayscale analysis schematic, it was clearly seen in Figure 1b that compared with the control group, the protein level of Collagen II in LPS-induced NPCs was sharply dropped. The above experiments indicate that LPS certainly caused severe cell viability damage to NPCs. Subsequently, KuA was added into NPCs at concentrations of 10, 20, 40, and 80 µM to observe its effect on cell viability. Figure 1c presented no noticeable change in cell viability as compared to the control group, while the results of the group with KuA concentration of 80 µM were not considered because of the cytotoxicity produced. Next, KuA at concentrations of 10, 20, and 40 µM were added to the LPS-induced NPCs. It was found in Figure 1d that cell viability increased in the LPS+KuA (10, 20, 40 µM) groups in a concentration-dependent manner (vs. LPS). Similarly, EdU experiment detected more EdU positive cells in the LPS+KuA (10, 20, 40 µM) groups than in the LPS group, and the number of EdU positive cells increased with the rise of KuA concentration (Figure 1e). Combining the above experimental results, it was concluded that KuA attenuated LPS-induced cell viability damage in NPCs.

(a) Cell viability in the groups of control, LPS (0.01, 0.1, 1, 10 µg/ml) was assessed by CCK-8. (b) Relative Collagen II protein expression in the control and LPS (1 µg/ml) groups was detected by western blot. (c) Cell viability in the groups of control, KuA (10, 20, 40, 80 µM) was assessed by CCK-8. (d) Cell viability in the groups of control, LPS, LPS+KuA (10, 20, 40 µM) was assessed by CCK-8. (e) EdU positive cells in the groups of control, LPS, LPS+KuA (10, 20, 40 µM) were assayed by EdU experiment. Results are the mean ± SD calculated from three independent experiments. **P < 0.01, ***P < 0.001 versus control. ^#^P < 0.05, ^##^P < 0.01, ^###^P < 0.001 versus LPS.

### KuA attenuates LPS-induced the apoptosis and ECM in NPCs

**Figure 2.**
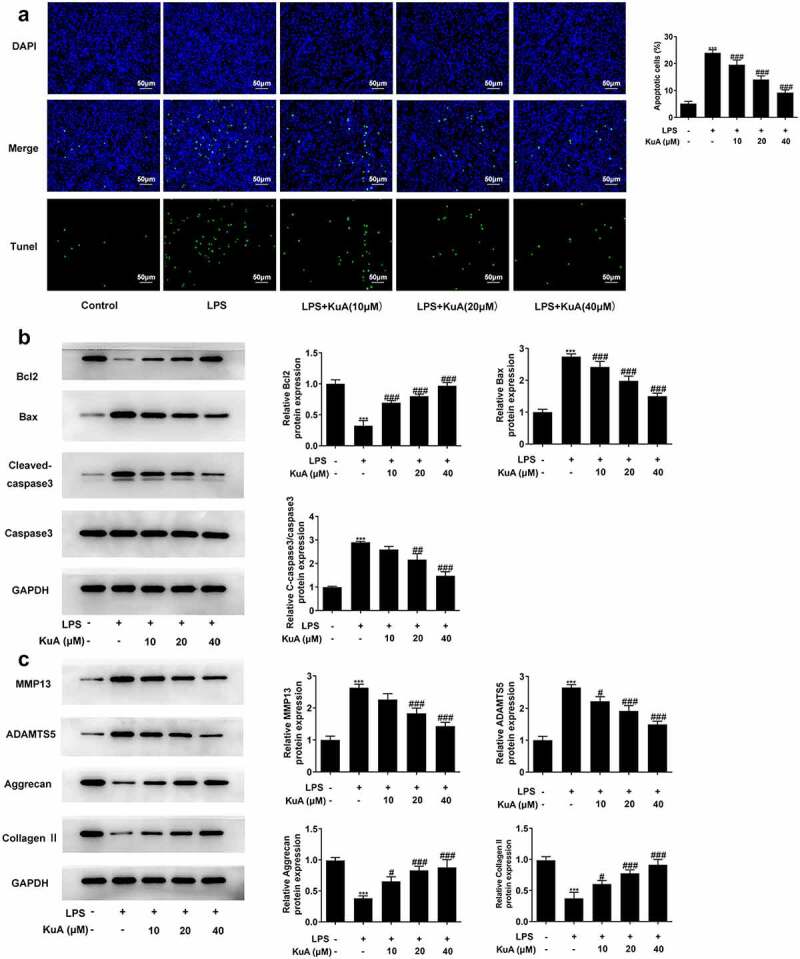
KuA attenuates LPS-induced apoptosis and ECM in NPCs.

To understand the impacts of KuA on the apoptosis and ECM in LPS-induced NPCs, TUNEL and western blot experiments were adopted in this part. As shown in Figure 2a, LPS induced a higher cell apoptotic rate (vs Control), while the number of apoptotic cells gradually decreased with the increase of KuA concentration. Moreover, the level of apoptosis-related protein Bcl-2 in NPCs reduced rapidly under the induction of LPS but elevated steadily with the rising concentration of KuA (Figure 2b). Not only that, the protein levels of Bax and C-cleaved 3 rose sharply in response to LPS induction but declined with growing KuA concentration. Furthermore, as indicated in Figure 2c, the protein levels of matrix metalloproteinase 13 (MMP13) and a disintegrin like and metalloproteinase with thrombospondin type I motifs 5 (ADAMTS5) were elevated in LPS-induced NPCs compared with the control group, but were steadily declined in the LPS+KuA (10, 20, 40 µM) groups. In addition, the protein levels of Aggrecan and Collagen II associated with cartilage degradation displayed a completely opposite trend to the two proteases mentioned above. To sum up, KuA could attenuate LPS-induced the apoptosis and ECM in NPCs.

(a) Apoptotic cells in the groups of control, LPS, LPS+KuA (10, 20, 40 µM) were calculated by TUNEL assay. (b) Protein levels of Bcl-2, Bax, C-cleaved 3 in the groups of control, LPS, LPS+KuA (10, 20, 40 µM) were detected by western blot. (c) Protein levels of MMP13, ADAMTS5, Aggrecan and Collagen II in the groups of control, LPS, LPS+KuA (10, 20, 40 µM) were determined by means of western blot. Results are the mean ± SD calculated from three independent experiments. ***P < 0.001 versus control. ^#^P < 0.05, ^##^P < 0.01, ^###^P < 0.001 versus LPS.

### KuA reduces LPS-induced inflammatory response in NPCs

**Figure 3.**
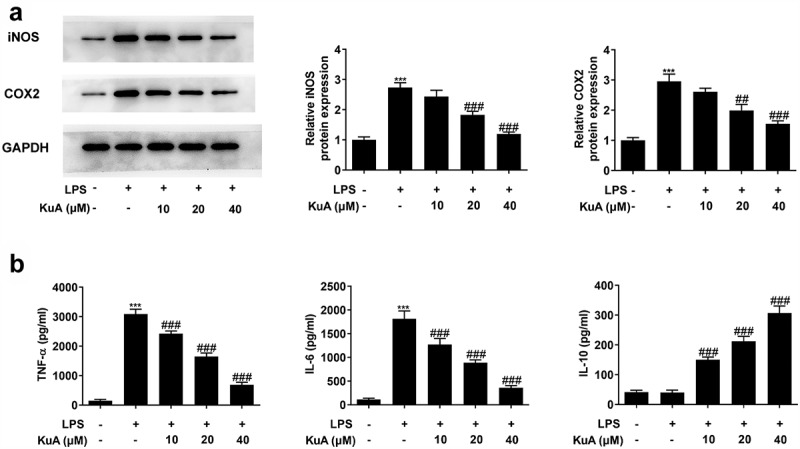
KuA reduces LPS-induced inflammatory response in NPCs.

To determine the effects of KuA on the inflammation in LPS-induced NPCs, the protein levels of iNOS and COX2 were assayed by western blot, and the levels of the inflammatory cytokines TNF-α, IL-6 and IL-10 by ELISA. As can be seen form the Figure 3a, the LPS group presented a significantly higher level of iNOS and COX2 than those of the control group. While the levels of iNOS and COX2 dropped gradually in the LPS+KuA (10, 20, 40 µM) groups. Additionally, Figure 3b-c showed the dramatic increase in the levels of TNF-α and IL-6 in the LPS group (vs Control), as well as the gradual decrease in levels of TNF-α and IL-6 in the LPS+KuA (10, 20, 40 µM) groups. From Figure 3d we can see that the LPS group exhibited similar level of IL-10 to the control group. However, KuA caused a dramatic increase in IL-10 levels in a concentration-dependent way. Based on the above analysis, KuA could reduce LPS-induced inflammatory response in NPCs.

(a) Protein levels of iNOS and COX2 in the groups of control, LPS, LPS+KuA (10, 20, 40 µM) were measured employing western blot. (b) Levels of TNF-α, IL-6 and IL-10 in the groups of control, LPS, LPS+KuA (10, 20, 40 µM) were tested by the way of ELISA assay. Results are the mean ± SD calculated from three independent experiments. ***P < 0.001 versus control. ^##^P < 0.01, ^###^P < 0.001 versus LPS.

### KuA activates the P13K/Akt pathway in LPS-induced NPCs

**Figure 4.**
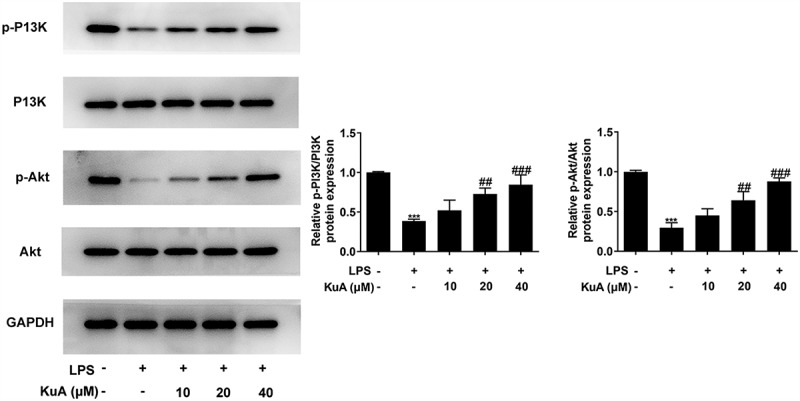
KuA activates the P13 K/Akt pathway in LPS-induced NPCs.

To verify whether KuA can activate the P13K/Akt pathway, the protein levels of p-P13K, P13K, p-Akt, and Akt were measured with the application with western blot. It can be seen in detail from Figure 4 that the protein levels of p-P13K and p-Akt were declined sharply in the LPS group (vs Control), but showed a concentration-dependent increase in the LPS+KuA (10, 20, 40 µM) groups. However, the protein levels of P13K and Akt remained unchanged in each group. These results suggested KuA could activate the P13K/Akt pathway in LPS-induced NPCs.

Protein levels of p-P13 K, P13 K, p-Akt and Akt in the groups of control, LPS and LPS+KuA (10, 20, 40 µM) were measured with the adoption of western blot. Results are the mean ± SD calculated from three independent experiments. ***P < 0.001 versus control. ^##^P < 0.01, ^###^P < 0.001 versus LPS.

### Inhibition of the P13K/Akt pathway reverses the promotion of proliferative capacity and the suppression of apoptosis by KuA in LPS-induced NPCs

**Figure 5.**
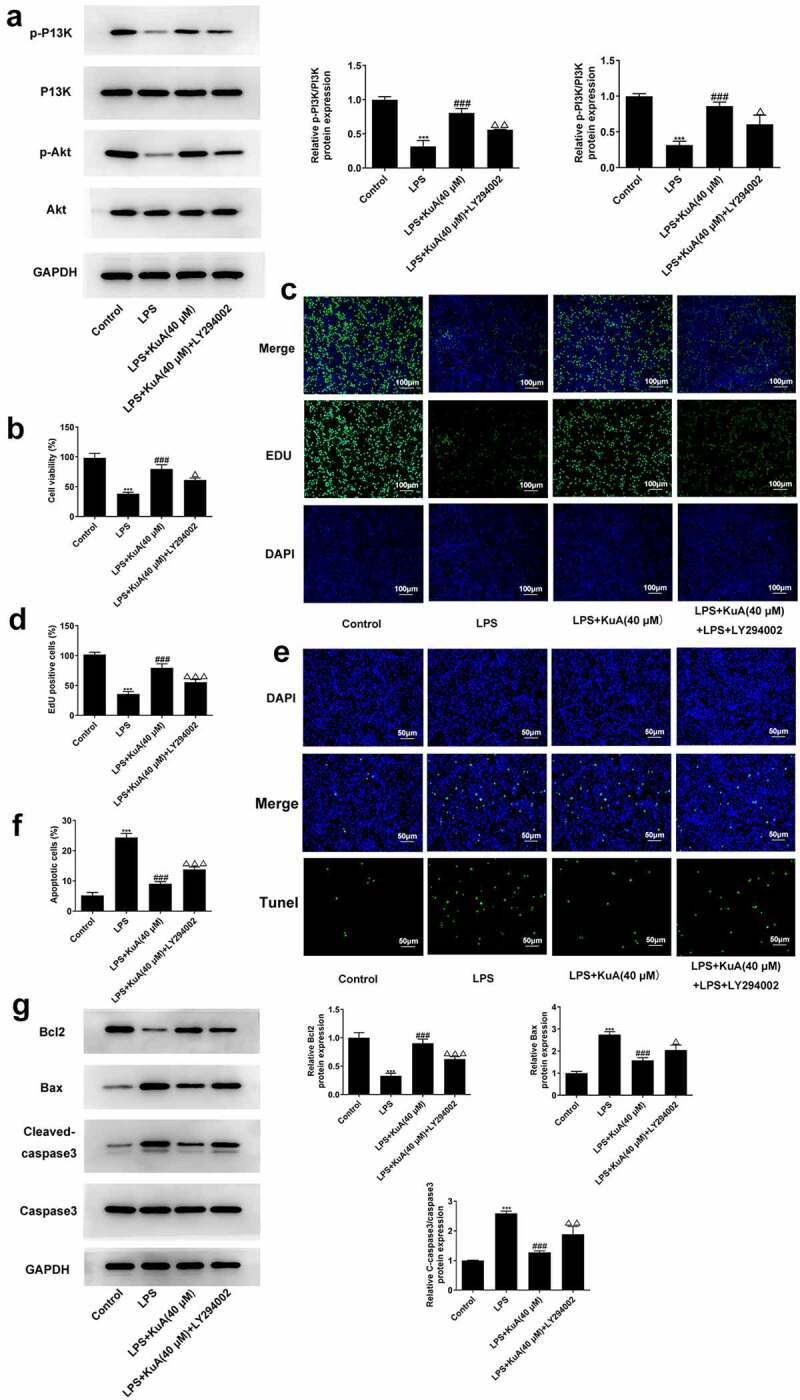
Inhibition of the P13 K/Akt pathway reverses the promotion of proliferative capacity and the suppression of apoptosis by KuA in LPS-induced NPCs.

The above experiment has confirmed that KuA could activate the P13K/Akt pathway in LPS-induced NPCs. To identify this result more closely, 25 μmol/L of P13 K/Akt pathway inhibitor LY294002 was added into the LPS+KuA (40 μM) group. LY294002 remarkably suppressed the activation of the P13 K/Akt pathway by KuA (Figure 5a). Additionally, it was previously demonstrated that KuA promoted the proliferation of LPS-induced NPCs and inhibited the apoptosis. Nevertheless, compared with the LPS+KuA (40 μM) group, cell viability in the LPS+KuA (40 μM) +LY294002 group was decreased (Figure 5b). EdU assay detected fewer EdU positive cells in the LPS+KuA (40 μM) +LY294002 group than those in the LPS+KuA (40 μM) group (Figure 5c). These two experimental results suggested that inhibition of the PI3K/Akt pathway reversed the promotive effects of KuA on the proliferation of LPS-induced NPCs. Next, TUNEL experiments showed an increase in the number of apoptotic cells in the LPS+KuA (40 μM) +LY294002 group in comparison with the LPS+KuA (40 μM) group (Figure 5d). Moreover, the levels of apoptosis-related protein Bcl-2 decreased again, while the levels of other two proteins Bax and C-cleaved 3 increased (Figure 5e). These experiments illustrated that inhibition of the PI3K/Akt pathway reversed the suppressive effects of KuA on the apoptosis of LPS-induced NPCs. Taken together, inhibition of the P13 K/Akt pathway reversed the promotion of proliferative capacity and the suppression of apoptosis by KuA in LPS-induced NPCs.

(a) Protein levels of p-P13 K, P13 K, p-Akt and Akt in the groups of control, LPS, LPS+KuA (40 µM) and LPS+KuA (40 µM) +LY294002 were detection by western blot. (b) Cell viability in the groups of control, LPS, LPS+KuA (40 µM) and LPS+KuA (40 µM) +LY294002 was assessed by CCK-8. (c) EdU positive cells were counted in the groups of control, LPS, LPS+KuA (40 µM) and LPS+KuA (40 µM) +LY294002 by EdU assay. (d) Apoptotic cells were calculated in the groups of control, LPS, LPS+KuA (40 µM) and LPS+KuA (40 µM) +LY294002 using TUNEL assay. (e) Protein levels of Bcl-2, Bax, C-cleaved 3 in the groups of control, LPS, LPS+KuA (40 µM) and LPS+KuA (40 µM) +LY294002 were detected by western blot. Results are the mean ± SD calculated from three independent experiments. ***P < 0.001 versus control. ^###^P < 0.001 versus LPS. ^Δ^P < 0.05, ^ΔΔ^P < 0.01, ^ΔΔΔ^P < 0.001 versus LPS++KuA (40 µM).

### Inhibition of the P13 K/Akt pathway reverses the attenuation of ECM and inflammation by KuA in LPS-induced NPCs

**Figure 6.**
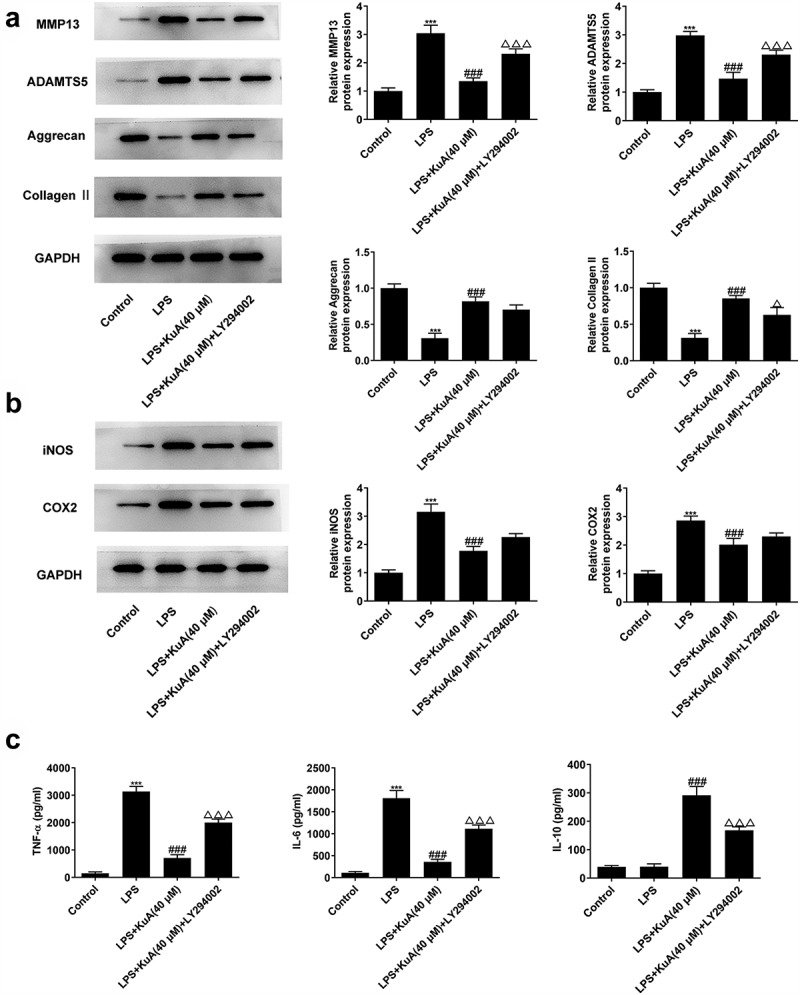
Inhibition of the P13 K/Akt pathway reverses the attenuation of ECM and inflammation by KuA in LPS-induced NPCs.

Previously, we have verified that KuA reduced ECM and inflammation in LPS-induced NPCs. Here, we wondered whether the inhibitory effect of KuA on LPS-induced ECM and inflammation in NPC was mediated by activation of the P13 K/Akt pathway. As shown in Figure 6a, LY294002 improved the protein levels of MMP13 and ADAMTS5, but reduced the protein levels of Aggrecan and Collagen II in LPS-induced NPCs after treatment of KuA (40 μM). Moreover, western blot in Figure 6b detected the elevated protein levels of iNOS and COX2 in the LPS+KuA (40 μM) +LY294002 group. Furthermore, compared with the LPS+KuA (40 μM) group, the increased levels of TNF-α and IL-6 in the LPS+KuA (40 μM) +LY294002 group was observed in Figure 6c–d. While LY294002 decreased the level of IL-10 in LPS-induced NPCs after treatment of KuA (40 μM) (Figure 6e). In general, inhibition of the P13 K/Akt pathway also reversed the attenuation of ECM and inflammation by KuA in LPS-induced NPCs.

(a) Protein levels of MMP13, ADAMTS5, Aggrecan and Collagen II in the groups of control, LPS, LPS+KuA (40 µM) and LPS+KuA (40 µM) +LY294002 were determined by western blot. (b) Protein levels of iNOS and COX2 in the groups of control, LPS, LPS+KuA (40 µM) and LPS+KuA (40 µM) +LY294002 were measured using western blot. (c) Levels of TNF-α, IL-6 and IL-10 in the groups of control, LPS, LPS+KuA (40 µM) and LPS+KuA (40 µM) +LY294002 were tested by means of ELISA. Results are the mean ± SD calculated from three independent experiments. ***P < 0.001 versus control. ^###^P < 0.001 versus LPS. ^Δ^P < 0.05, ^ΔΔΔ^P < 0.001 versus LPS++KuA (40 µM).

## Discussion

Intervertebral disc degeneration (IDD) is the basis for many musculoskeletal and spinal disorders and is also a major cause of low back pain [1]. Based on Clouet., et al., extracellular matrix degeneration, inflammation and cell apoptosis are the most widespread pathological changes in IDD [6]. Previously, KuA was shown to have anti-inflammatory and anti-apoptotic effects and to activate the P13 K/Akt pathway. This paper discussed the effects of KuA on IDD from three perspectives: apoptosis, extracellular matrix degradation (ECM), and inflammation. The current study in this paper found that KuA could attenuate LPS-induced cell damage, apoptosis, ECM, and inflammation in NPCs. Moreover, another important finding was that KuA activated the P13 K/Akt pathway in LPS-induced NPCs. However, the addition of P13 K/Akt pathway inhibitors LY294002 reversed the effects of KuA on LPS-induced NPCs. These results further supported the idea that KuA attenuated LPS-induced apoptosis, ECM and inflammation in NPCs by activating the P13 K/Akt pathway.

Increased cellular senescence and death, altered phenotype of healthy disc cells and reduced number of active cells are considered to be among the pathological features of IDD [26]. The process of IDD leads to the accumulation of cellular waste and degraded matrix molecules, which creates an acidic environment that further impairs cell viability [27]. In this study, LPS induced a lower cell viability in NPCs. However, KuA significantly attenuated LPS-induced cell viability impairment of NPCs. Additionally, excessive apoptosis of cells leads to a decrease in the number of NPCs, which in turn leads to a decrease in the synthesis of ECM, thereby contributing to the degeneration of IDD [11]. Previous studies have shown that degenerate NP cells have more apoptotic cells than normal controls [28]. In our study, LPS induced more apoptotic cells compared than the control group, which was in agreement with those of earlier studies. Additionally, KuA poses anti-oxidative effect and anti-apoptosis in vitro. For example, KuA could protein pMCAO-induced brain injury by mitochondria mediated apoptosis signaling pathway [29]. KuA protects against neurotoxin-induced Parkinson’s disease by inhibiting apoptosis and enhancing autophagy [30]. In this paper, gradually reduced apoptotic cells were found in the groups of LPS+KuA (10, 20, 40 µM). Moreover, KuA also regulates apoptosis-associated proteins [21]. Bcl-2 is known to be a mitochondria-associated anti-apoptotic protein. Our experiments detected a progressive increase in protein levels of Bcl-2 in the groups of LPS+KuA (10, 20, 40 µM), which revealed the fact that KuA could protect against LPS-induced apoptosis of NPCs in IDD.

The physiological function of the intervertebral disc is maintained by extracellular matrix produced by the nucleus pulposus cells [31]. When the balance between extracellular matrix synthesis and degradation is disrupted, it leads to structural failure and biomechanical changes in IDD [32]. MMP and ADAMTS have been reported to mediate matrix remodeling in NPCs [33,34]. In addition, the extracellular matrix of NPCs is composed of proteoglycans and collagen, with aggrecan being the most abundant proteoglycan in the nucleus [35]. However, with increasing degeneration, the expression of proteoglycans and collagen II gradually decreases [36]. At the same time, there is an increasing secretion of ECM molecules, such as MMP and ADAMTS [34]. In our study, it was clearly observed in the groups of LPS+KuA (10, 20, 40 µM) that there were the declined protein levels of MMP13 and ADAMTS5, as well as elevated protein levels of Aggrecan and Collagen II. These results suggested that KuA attenuated ECM in LPS-induced NPCs.

iNOS and COX-2 are important inflammatory mediators in the pathogenesis of IDD, inappropriate upregulation of iNOS and COX-2 is associated with the pathophysiology of inflammatory diseases [37]. In our study, the levels of iNOS and COX2 rose in LPS-induced NPCs but dropped after treatment of KuA. On the other hand, extracellular matrix catabolic products produced during disc degeneration may promote macrophage-mediated IL-1β and tumor necrosis factor α (TNF-α) production through activation of NF-κB/IκBα complexes [38]. Meanwhile, the levels of IL-6 and IL-17 were elevated [3]. TNF-α was demonstrated to promote intervertebral disc breakdown and induce a series of pathogenic reactions in NPCs, promoting autophagy, senescence and apoptosis [39]. Moreover, it has been evidenced that an increase in IL-6, IL-8 and PGE2 was observed in control and degenerated human IVD tissues in response to lipopolysaccharide (LPS) stimulation [40]. Our experiments showed a decrease in Pro-inflammatory cytokines TNF-α and IL-6 and an increase in anti-inflammatory mediator IL-10 in the groups of LPS+KuA (10, 20, 40 µM). These results implied that KuA alleviates LPS-induced inflammation in NPCs.

On the other hand, PI3K/Akt is involved in regulating oxidative stress and inflammatory responses through Nrf2 [41]. It has been reported that inhibition of PI3K/Akt signaling pathway activity leads to reduced proteoglycan matrix deposition and reduced aggrecan gene expression in NPCs [42]. In our study, western blot detected reduced levels of p-P13k and p-Akt in LPS-induced NPCs as well as progressively increased levels of P13 K and Akt in the groups of LPS+KuA (10, 20, 40 µM), which suggested that KuA activated PI3K/Akt pathway. However, subsequent experiments showed that treatment with the P13k/Akt inhibitor LY294002 partially reversed the role of KuA in LPS-induced NPCs. Here, there are several limitation in our study. KuA concentration used in this study is different from that in animals and human, thus the result of this study might not be suitable for in vivo experiments and clinical trials. In addition, although LPS stimulated NPC are a valid model, they do not directly represent a degenerated IVD environment. Thus, we will perform *in vivo* experiments and clinical trials to confirm the effects of KuA in IDD in further study.

## Conclusion

To sum up, this study offers clear evidence for the role of KuA in LPS-induced NPCs. That is, KuA can reduce LPS-induced apoptosis, ECM and inflammation in NPCs by activating the PI3K/Akt pathway. These findings suggested that KuA may serve as a novel therapeutic strategy and alternative drug for IDD.
